# Use of the English urgent referral pathway for suspected cancer and mortality in patients with cancer: cohort study

**DOI:** 10.1136/bmj.h5102

**Published:** 2015-10-14

**Authors:** Henrik Møller, Carolynn Gildea, David Meechan, Greg Rubin, Thomas Round, Peter Vedsted

**Affiliations:** 1 Cancer Epidemiology and Population Health, King’s College London, London SE1 9RT, UK; 2 Public Health England, Knowledge & Intelligence Team (East Midlands), Sheffield, UK; 3 School of Medicine, Pharmacy and Health, University of Durham, Stockton on Tees, UK; 4 Division of Health and Social Care, King’s College London; 5 Research Unit for General Practice, Centre for Cancer Diagnosis in Primary Care, Department of Public Health, Aarhus University, Aarhus, Denmark

## Abstract

**Objective** To assess the overall effect of the English urgent referral pathway on cancer survival.

**Setting** 8049 general practices in England.

**Design** Cohort study. Linked information from the national Cancer Waiting Times database, NHS Exeter database, and National Cancer Register was used to estimate mortality in patients in relation to the propensity of their general practice to use the urgent referral pathway.

**Participants** 215 284 patients with cancer, diagnosed or first treated in England in 2009 and followed up to 2013.

**Outcome measure** Hazard ratios for death from any cause, as estimated from a Cox proportional hazards regression.

**Results** During four years of follow-up, 91 620 deaths occurred, of which 51 606 (56%) occurred within the first year after diagnosis. Two measures of the propensity to use urgent referral, the standardised referral ratio and the detection rate, were associated with reduced mortality. The hazard ratio for the combination of high referral ratio and high detection rate was 0.96 (95% confidence interval 0.94 to 0.99), applying to 16% (n=34 758) of the study population. Patients with cancer who were registered with general practices with the lowest use of urgent referral had an excess mortality (hazard ratio 1.07 (95% confidence interval 1.05 to 1.08); 37% (n=79 416) of the study population). The comparator group for these two hazard ratios was the remaining 47% (n=101 110) of the study population. This result in mortality was consistent for different types of cancer (apart from breast cancer) and with other stratifications of the dataset, and was not sensitive to adjustment for potential confounders and other details of the statistical model.

**Conclusions** Use of the urgent referral pathway could be efficacious. General practices that consistently have a low propensity to use urgent referrals could consider increasing the use of this pathway to improve the survival of their patients with cancer.

## Introduction

Achieving an earlier diagnosis of cancer at a less advanced stage is a public concern and has become a widely adopted priority for healthcare systems. It is generally assumed that the more promptly a diagnosis of cancer is made, the better is the prognosis, because cancer detected at an earlier stage has better treatment options leading to improved survival.^1^ This assumption is supported by evidence that tumours can progress during the time taken to reach a diagnosis and start treatment.^2^ ^3^ Observational studies have found an association between time to diagnosis and mortality.^4^ ^5^ ^6^ ^7^

The time for a patient to reach a diagnosis of cancer can be influenced by patient, practitioner, and healthcare system delays.^8^ Several countries where general practitioners have a gatekeeping role for access to specialist care have introduced (or are introducing) urgent referral pathways for patients with symptoms suggestive of cancer.^9^ ^10^ ^11^ Typically, such pathways enable rapid access to a specialist opinion or diagnostic test (within two weeks in England) for patients with specified alarm symptoms, such as abnormal bleeding, unexplained weight loss, or an unexplained lump or abdominal mass. These pathways are supported by clinical guidelines for their use. 

The current pathway in England^12^ was revised in June 2015. For some cancers, evidence now suggests that use of the pathway is associated with a shorter time to diagnosis and treatment, although the size of the effect varies by cancer site.^8^ ^13^ ^14^ ^15^ ^16^ ^17^ ^18^ For colorectal cancer, in one report from a Spanish research group, time to diagnosis after urgent referral was 19 days less than by routine referral.^16^ However, there is not yet evidence indicating that this diagnostic strategy has an effect on the overall prognosis and mortality of patients with cancer in the population.

In the United Kingdom, the frequency with which general practices use the urgent referral pathway for suspected cancer and the accuracy of their patient selection for urgent referral varies considerably.^19^ ^20^ ^21^ This variation provides an opportunity to explore and quantify the association between use of urgent referral and cancer outcomes. We assessed the association between the propensity of general practices to use urgent referral and the precision of its use, and the overall mortality among their patients with cancer.

## Methods

Our analysis used a dataset of urgent referrals and cancer diagnoses constructed by Meechan and colleagues^20^ from the English national Cancer Waiting Times^22^ database, where information was collected from trusts in the UK’s health service for official monitoring of the Cancer Waiting Times standards. This dataset, of patients with a date of a first hospital appointment or treatment in 2009, contained records of 865 494 urgent referrals for suspected cancer and 224 984 diagnosed cancers from 8049 general practices; 43% of the cancers were diagnosed following urgent referral. To associate each patient with a general practice, these records were linked by NHS number, through the open Exeter portal tracing service, to the NHS Exeter database.^23^ The NHS Exeter database contains registration details for all patients registered with an NHS general practitioner in England and Wales; Cancer Waiting Times records that were not linked here were not included in the Meechan and colleagues’^20^ dataset.

For verification of the cancer diagnosis and survival follow-up, the records were also linked with the National Cancer Register^24^ ^25^ ^26^ by NHS number, date of diagnosis, and cancer type. We excluded from all analyses the small proportion of patients for whom there was no verified diagnosis.

We also excluded practices with a very small list size or a large change in list size.^20^ All patients in the study had non-missing values for all variables, except 141 patients who had missing data for socioeconomic status. In the sensitivity analysis with adjustment for socioeconomic status, these 141 patients were included as a separate group.

We used three referral metrics:

- Practice referral ratio: the indirectly standardised number of urgent referrals for suspected cancer by general practitioners, standardised according to the general practice’s list size and to the age and sex distributions of people on the list. This number was computed by the ratio of the observed number of referrals to the expected number of referrals (with the expected number calculated from age and sex specific list size data for each general practice, and from national age and sex specific rates of urgent referral)
- Practice conversion rate: the proportion of urgent referrals for suspected cancer by general practitioners that result in a diagnosis of cancer. This is the positive predictive value for cancer among the patients selected for urgent referral
- Practice detection rate: the proportion of Cancer Waiting Times recorded cancers resulting from an urgent referral for suspected cancer by general practitioners. This is the sensitivity of the selection of patients for urgent referral in the general practice.

We used Cox proportional hazards regression to analyse and quantify the hazard of death from any cause in relation to referral ratio, conversion rate, and detection rate; we assessed these measures individually, conditionally (mutually adjusted), and with stratification by combinations. All analyses were adjusted for age (linear and second order terms) and sex (binary) of the individual patient, as well as for the main types of cancer (colorectal, lung, breast, prostate, other) as a categorical variable. The main analysis used the four year time window of follow-up from diagnosis to death, censoring at four years or at the study end date in December 2013, whichever occurred first.

We used Schoenfeld residuals to explore the assumption of proportional hazards. The assumption was generally upheld, but there were some differences between the main types of cancer (web appendix B-G).

Referral ratio, conversion rate, and detection rate were analysed as categorical variables, according to groups divided by tertiles of their distributions (that is, divided into three equal groups). Analysis by categorical variables was decided a priori because we expected that associations might be non-linear, and in order to facilitate analysis of the joint effects of the variables.

We pursued various stratified analyses and sensitivity analyses to improve the consistency and internal validity of the findings. The main analysis used a four year follow-up, and we also explored shorter durations of follow-up by censoring at one or two years after diagnosis. We checked the sensitivity of the principal findings in the subset of patients for whom diagnosis and date of diagnosis were consistent with the National Cancer Register (that is, identical ICD-10 diagnosis codes at the three digit level, and dates of diagnosis within 90 days of each other). 

We analysed the data for each of the most frequent types of cancer separately, and evaluated whether the overall results changed if no adjustment was made for cancer type. In separate analyses, we adjusted for the patient’s socioeconomic status or for the age and sex distribution of the general practice’s list population. For socioeconomic status, we used groups based on quintiles of the income domain of the index of multiple deprivation as a categorical variable (that is, divided into five equal groups). For age and sex distribution, we used groups based on tertiles of the median age of patients from each practice as a categorical variable (that is, divided into three equal groups), and the proportion of male patients on the list as a continuous variable. We used a shared frailty random effects model^27^ to accommodate the multilevel structure of data where groups of patients with cancer belong to the same general practice list. All analyses were carried out with Stata version 12.^28^

### Patient involvement

No patients were involved in setting the research question or the outcome measures, nor were they involved in the design and implementation of the study. Study results and implications were discussed with patients with cancer, carers and lay people at meetings, workshops and conferences. The work on cancer diagnostics in King’s College London is overseen by a patient and public involvement steering group.

## Results

The cohort of patients with cancer for analysis, after elimination of multiple records (5276 records) and patients for whom there was no verified diagnosis in the National Cancer Register (4374 patients), comprised 215 284 patients with cancer (fig 1). During four years of follow-up, 91 620 deaths occurred, of which 51 606 (56%) occurred within the first year after diagnosis.

**Figure fig1:**
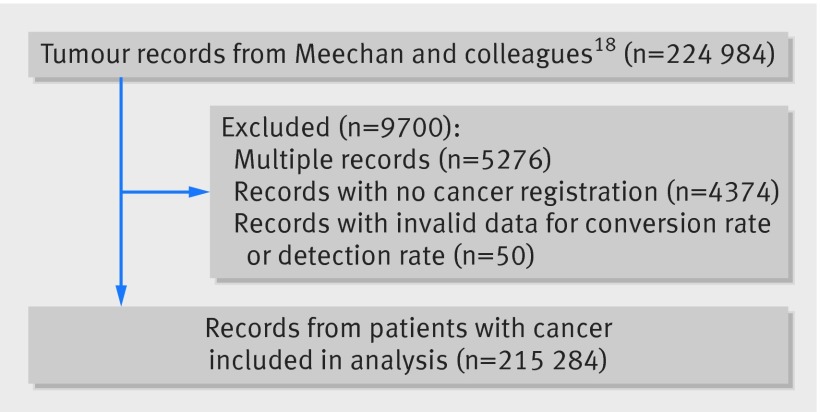
**Fig 1** Flow diagram of study population with inclusions and exclusions

Figure 2 shows the distributions of referral ratio, conversion rate, and detection rate. The three measures were moderately correlated. Correlation coefficients were: referral ratio versus detection rate (0.42), referral ratio versus conversion rate (−0.35), and detection rate versus conversion rate (0.37). The table[Table tbl1] shows the analysis of all cause mortality in relation to referral ratio, conversion rate, and detection rate. Each distribution was divided by tertiles (that is, in three equal groups). Referral ratio was associated with mortality, with hazard ratios of 1.05 (95% confidence interval 1.04 to 1.07) in the lowest group and 0.97 (0.96 to 0.99) in the highest group, each compared with the intermediate group. Detection rate was also associated with mortality, with hazard ratios of 1.04 (1.02 to 1.06) in the lowest group and 0.96 (0.95 to 0.98) in the highest group. Conversion rate was not associated with mortality.

**Figure fig2:**
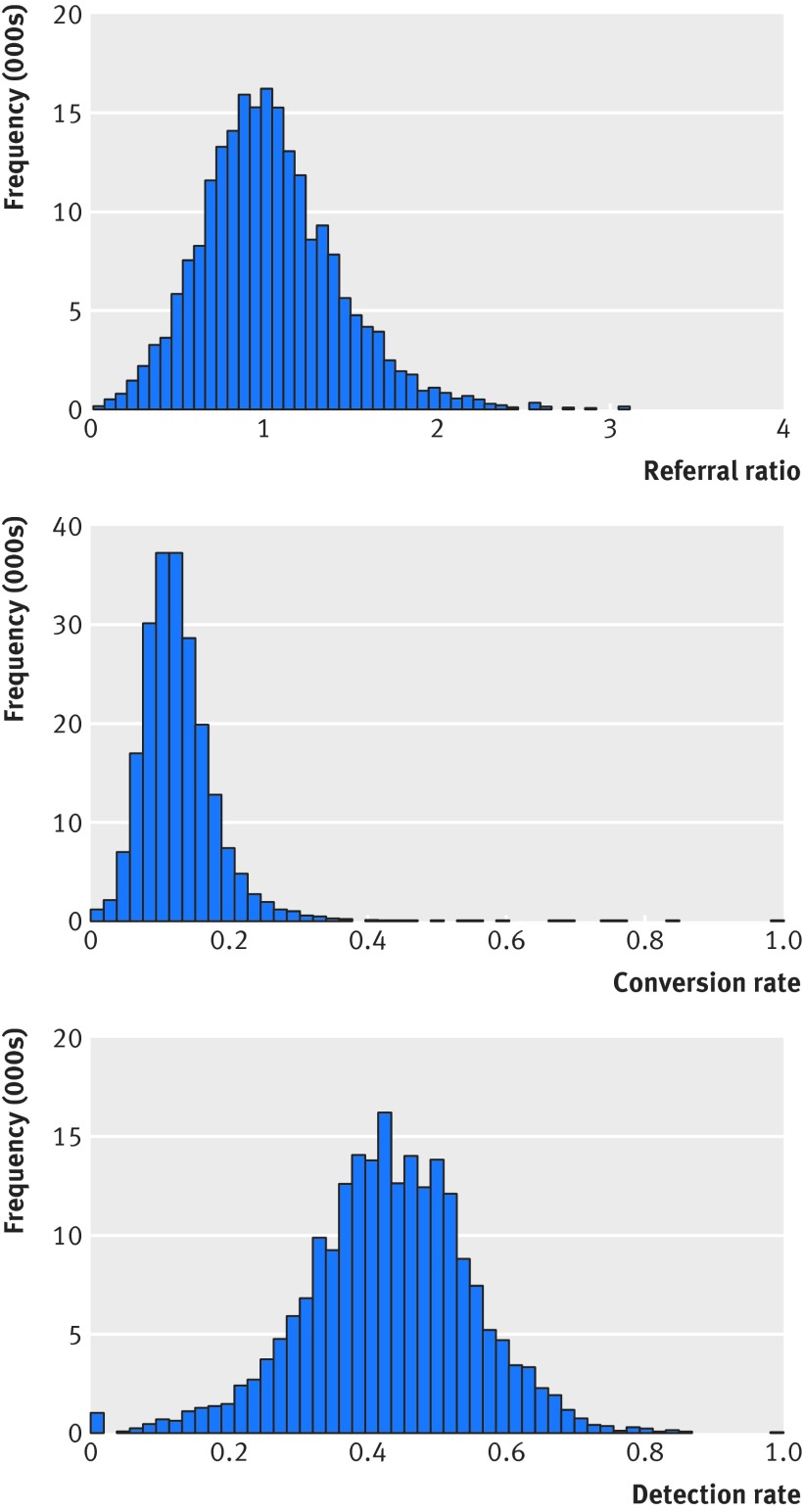
**Fig 2** Histograms of referral ratio, conversion rate, and detection rate

**Table tbl1:** Hazard ratios for death in relation to referral ratio, conversion rate, and detection rate

Variable and group	Median value	Lowest value	Highest value	No of people	No of deaths	Hazard ratio (95% CI)*	Hazard ratio (95% CI)†
**Referral ratio**							
Low	0.68	0.20	0.86	71 773	31 136	1.05 (1.04 to 1.07)	1.04 (1.02 to 1.06)
Intermediate	1.01	0.86	1.16	71 768	30 417	1.00	1.00
High	1.39	1.16	3.44	71 743	30 067	0.97 (0.96 to 0.99)	0.98 (0.97 to 1.00)
P_trend_	—	—	—	—	—	<0.001	<0.001
**Conversion rate**							
Low	0.08	0.00	0.10	71 811	30 206	1.00 (0.99 to 1.02)	1.00 (0.98 to 1.02)
Intermediate	0.12	0.10	0.14	72 101	30 672	1.00	1.00
High	0.17	0.14	1.00	71 372	30 742	1.01 (1.00 to 1.03)	1.00 (0.98 to 1.02)
P_trend_	—	—	—	—	—	0.117	0.748
**Detection rate**							
Low	0.33	0.00	0.39	71 804	31 072	1.04 (1.02 to 1.06)	1.03 (1.01 to 1.05)
Intermediate	0.43	0.39	0.48	72 065	30 749	1.00	1.00
High	0.54	0.48	1.00	71 415	29 799	0.96 (0.95 to 0.98)	0.97 (0.95 to 0.99)
P_trend_	—	—	—	—	—	<0.001	<0.001

*Adjusted for age, sex, and cancer type.

†Adjusted for age, sex, and cancer type; and mutually adjusted.

The web figure shows the analyses based on groups divided by deciles of the distributions of referral ratio, conversion rate, and detection rate (that is, divided into 10 equal groups). Mortality decreased with the level of referral ratio and detection rate. There was no suggestion of attenuation of the effects of referral ratio and detection rate towards the highest groups. We saw no suggestion of an association with conversion rate, even at the lowest or highest groups.

Figure 3 shows the detailed analysis of joint effects of referral ratio and detection rate on mortality, with each measure divided into three equal groups. There are nine combinations in this 3×3 classification, and we used the intermediate groups of the two variables as the reference category in the Cox regression analysis. A low hazard ratio was seen for the combination of high referral ratio and high detection rate (0.96 (95% confidence interval 0.94 to 0.99); hereafter referred to as group 1 of the study population). High hazard ratios were seen for combinations of low referral ratio and low detection rate, or if one variable was low and the other was intermediate. The corresponding hazard ratios for these combinations were 1.08 (1.06 to 1.11), 1.07 (1.04 to 1.10), and 1.05 (1.02 to 1.08); we refer to this segment of the study population as group 3. Group 2 refers to the remaining study population.

**Figure fig3:**
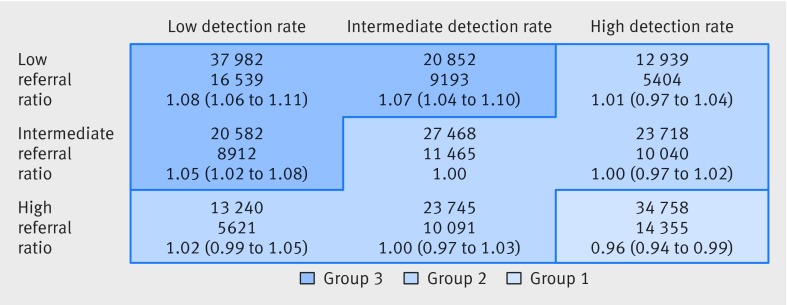
**Fig 3** Hazard ratios for death in two way classification of referral ratio and detection rate. Data are numbers of people and deaths, and adjusted hazard ratios for death with 95% confidence intervals

Figure 3 shows combinations of referral ratio and detection rate with similar hazard ratios. Group 1 (the bottom right section of figure 3) comprised 16% (n=34 758) of the study population with a high referral ratio and high detection rate (hazard ratio 0.96 (95% confidence interval 0.94 to 0.98), and group 3 (the top left section of figure 3) comprised 37% (n=79 416) of the study population with a low or low/intermediate referral ratio and detection rate (1.07 (1.05 to 1.08)). These groups were compared with group 2, which comprised 47% (n=101 110) of the study population, had other combinations of referral ratio and detection rate, and had hazard ratios close to 1. 

To give illustrate the absolute magnitude of the observed differences, we derived the cumulative mortality at four years from the Cox regression models. The extreme difference between patient groups in the 3×3 classification in figure 3 was a 6% point difference in four year survival, with the hazard ratios of 1.08 and 0.96 corresponding to cumulative mortality risks of 53% and 47%, respectively. For the three derived groups (with hazard ratios of 1.07, 1.00, and 0.96, respectively) the corresponding cumulative risks were 52%, 49%, and 47%. The 3% point difference in four year cumulative mortality between group 3 (corresponding to a low use of urgent referral) and group 2 (corresponding to the more common, higher use of urgent referral) applies to almost 80 000 patients in group 3. This difference suggests that an additional 2400 patients with cancer in group 3 might have been alive at the four year time point if the use of urgent referral had been higher, as in group 2.

The web appendix shows stratified analyses and sensitivity analyses of the three groups identified from figure 3. The general pattern was observed consistently for the main cancer types, with the exception of female breast cancer, which had no association with mortality across the three groups**.** Exclusion of breast cancer from the overall analysis did not change the general result. Compared to the overall analysis with up to four years of follow-up, there were slightly stronger mortality effects in the first year of follow-up. 

The overall results did not change materially when adjusted for the patient’s socioeconomic status, or restricted to the subset of patients for whom the diagnosis and date of diagnosis were consistent with the cancer registry. Results remained unchanged after we made no adjustment for type of cancer, or with additional adjustment for characteristics of the general practice list population. Furthermore, we fitted a random effects model to accommodate the multilevel structure of data according to groups of patients with cancer belonging to each practice list, which made no effect on the overall results.

## Discussion

### Principal findings

This analysis of the mortality of more than 200 000 patients with cancer in England shows that the propensity of general practices to use the urgent referral pathway for suspected cancer was associated with the mortality outcome of their patients. Their accuracy in case selection for urgent referral (conversion rate), however, was not associated with mortality. Referral ratio and detection rate are both measures of a general practice’s propensity to use the urgent referral pathway, and both were independently associated with mortality. The hazard of death was 4% lower for the 16% of patients from practices with high use and 7% higher for the 37% of patients from practices with low use, compared with the 47% of all patients from practices with intermediate use.

### Interpretation and comparison with other research

The association between use of the urgent referral pathway and mortality is consistent for the main types of cancer except breast cancer and not sensitive to details of inclusion or exclusion of patient subgroups or to the details of the statistical model of analysis. Thus, there is most plausibly an underpinning principle or mechanism of a general nature, although the possibility of bias from unknown confounding variables cannot be ruled out.

The associations with mortality were stronger in the first year after diagnosis, which may point to a role of cancer stage in the underlying mechanism. A reduction in diagnostic interval could contribute towards lower mortality through a more favourable stage distribution and better treatment options.

One important finding was that the conversion rate was not associated with mortality. Based on extensive research on the positive predictive values of symptoms that may suggest cancer in general practice,^29^ ^30^ there is a prevailing assumption that a high conversion rate is a consequence of general practitioners referring mainly when the patient has developed alarm symptoms for cancer. In turn, this point of referral is associated with higher mortality rates for their patients. The lack of association in the present analysis could reflect the clinical assessment made by the general practitioner when deciding on referral. Referral decisions involve a multifactorial process, taking account of a patient’s demographic features, medical history, help seeking behaviour, and preferences, rather than simply responding to symptoms of possible cancer and relating these to guidelines. The observed effect of urgent referral could therefore reflect general practitioners’ cancer awareness and willingness to use the urgent referral pathway for suspected cancer rather than the adherence to specific symptom based guidelines.

Indirect effects should be considered in this observational study, such as the possible effect of the lead time of earlier diagnoses on estimated mortality hazard ratios.^31^ An earlier diagnosis would inevitably reduce estimated death rates of patients with cancer, even without any effect on stage distribution or prognosis.

There could be other factors associated with general practices with a high referral ratio and high detection rate that account for the observed lower mortality. For example, general practitioners in practices with older patients could be more aware of cancer and more familiar with the urgent referral pathway. This might contribute to the finding in sensitivity analyses of an association between the age composition of the practice population and the mortality of patients with cancer (that is, higher age is associated with lower mortality), independent of the effect of referral ratio and detection rate.

In a study^32^ using data from 2012 on referral and cancer stage, higher use of urgent referral of patients with suspected cancer was associated with a reduced proportion of patients with advanced cancer. The study had insufficient follow-up time to consider the survival outcome, but it supports our interpretation that high use of the urgent referral pathway could operate through a stage mediated mechanism, as opposed to a lead time effect. It remains a weakness of our data that stage was poorly recorded in 2009. Since the modernisation of the English cancer registry, the recording of stage has been much improved and we will be able to analyse the mediation effect of stage on survival when the 2012 data has accrued a few more years of follow-up for death.

Other potential indirect mechanisms are that where general practitioners have better access to pre-referral tests, they may use these tests more frequently in patients with suspicious symptoms, identifying those with cancer earlier while also being more accurate in their case selection for urgent referral. Variation in the quality of clinical care could mean that patients receiving less good quality care could have diagnoses later, more frequently after emergency presentation, and have worse outcomes. Furthermore, our analyses assume that specialist service provision and treatment decision making is comparable for patients of all practices. Although national guidance has existed for some time on the specialist diagnosis and management of individual cancers, a degree of variation in clinical practice of individual specialists is inevitable.

In breast cancer, there was no association with referral ratio or detection rate, which is a departure from the overall pattern. The availability of the organised mammographic screening programme could result in many patients with early stage (and therefore good prognosis) breast cancer being detected through screening and not through the urgent referral pathway. We attempted to explore this idea by analysing cervical cancer, where an organised screening programme also exists, but the numbers of deaths were low and the results not conclusive. In the English urgent referral system, the inclusion criteria have been extended to ensure that women with breast symptoms who do not meet the clinical criteria for suspected cancer are still seen within two weeks. For patients with breast cancer, this would reduce the differences between general practices’ low and high referral ratios.

### Strengths and limitations of the study

The analysis was based on the complete national population of England, using nearly complete Cancer Waiting Times records from hospitals and cancer incidence and death records from population based cancer registration. The analysis strategy aimed to create the same result as that from an intention to treat analysis of a controlled trial of urgent referral. We included all patients with cancer in the mortality analysis and hereby eliminated some known biases and artefacts that arise from two things:

- A comparison of patients with cancer with short and long time to treatment (the waiting times paradox,^33^ where short delay has been associated with poor prognosis through reverse causation)
- Comparison of urgently and non-urgently referred patients, subject to selection bias and confounding by indication, and often with different results for different types of cancer and different red flag symptoms.^34^ ^35^

If urgent referral was reserved only for those patients presenting alarming symptoms that might indicate advanced cancer stage, a higher use of urgent referral could be biased towards a less favourable outcome (increased mortality). Nevertheless, we were able to demonstrate the opposite result of lower mortality associated with higher use of urgent referral.

By its design, the present analysis attempted to determine whether there is a mortality difference between a situation where an urgent referral route exists and is being used and another situation where such a mechanism does not exist or is not being used. Because the effects on mortality were estimated by time to event, there would be a contribution of lead time to the observed effect on mortality. We were not able to correct for this, or estimate the contribution of lead time from these data. Based on data from the UK General Practice Research Database, it was found that the introduction of guidelines for urgent referral from the National Institute for Health and Care Excellence in 2005 led to an average reduction in diagnostic interval of 5.4 days.^36^ A lead time of this magnitude is not likely to fully explain the observed mortality differences in the present analysis. However, the present analysis was able to look at the most likely causes of possible case mix variation between the groups of general practices (age, sex, types of cancer, and socioeconomic group), but as in any observational study, there remains, in principle, the possibility of bias from unknown confounding variables.

At the level of an individual practice, the measured characteristics (referral ratio, conversion rate, and detection rate) are often based on small numbers of referrals and cancer cases, and it should be appreciated that the measurements are inherently variable, including year on year random variation and cancer case mix.^21^ We advise against over-interpretation of these results at the level of the individual general practitioner or practice in a single year, but we believe that the identified patterns—based on aggregate groups of general practices and their patient populations—contain relevant and important information. As a consequence of the inherent variability of these measurements, the magnitude of the estimated mortality effect is likely to be under-estimated through non-differential misclassification. The joint effect of referral ratio and detection rate, where these are consistent (that is, both are low or both are high), is a repeated measurements situation that may identify general practices more accurately with a truly low or a truly high propensity to use urgent referral.^37^

In the present analysis, we calculated the referral ratio, conversion rate, and detection rate in a single year’s activity and for all cancers combined. Although we found consistent results for the main types of cancer (except breast cancer) and the results were not sensitive to the adjustment for cancer type, cancer awareness and choice of referral mechanism might not be the same for all cancer types and their symptoms. A more focused analysis could start with characteristics of urgent referral computed separately for each of the main types of cancer. This would require analysis based on several years of data and also, optimally, information on symptoms.

### Questions arising from this study

The analysis and interpretation of the results have led us to define new questions for future studies. Firstly, it should be established whether the general association between use of urgent referral and mortality of patients with cancer can be replicated in a more recent period, and in other national populations with comparable referral pathways. Secondly, it is necessary to understand how stable practice referral and detection rates are, year on year. Thirdly, the characteristics of practices with low referral and detection rates merit further study. If these measures are associated more generally with other measures of practice performance, interventions and education could be more targeted. Fourthly, the health economic consequences of modifying the practice referral ratio need to be elaborated. For some practices, the use of urgent referral might be too low and increased use in the group of general practices with both low referral ratio and low detection rate could lead to further improvement in cancer survival. 

We are aware that the increased use of urgent referral could be costly in terms of diagnostic and clinical resource, and consider that the cost effectiveness of increasing activity within the urgent referral pathway for suspected cancer should be explored in detail. Since 2009, the use of urgent referral in England has increased by more than 50% to over 1.2 million referrals per year.^38^ Updated NICE guidance on urgent referral for suspected cancer lowers the threshold for referral,^12^ ^39^ and has been modelled to potentially increase referrals to two million per year.^40^ To ensure the most rational use of urgent referral, its use must be based on sound clinical research conducted in general practice and take account of available guidelines. We should continue to monitor the variability in the use of this referral pathway and its association with mortality.

### Conclusion and implications

In this analysis, we have found a clinically relevant association between the low use of urgent referral for suspected cancer in general practices and increased risk of death among patients with cancer. The conversion rate of urgent referrals to cancer diagnoses was not associated with mortality. This lack of association suggests that the general practitioners’ cancer awareness and willingness to use the urgent referral pathway, together with the complete clinical assessment, could produce a positive effect of urgent referral for suspected cancer.

We have identified a subgroup of 37% of patients with cancer who were registered in 2009 with general practices with a low propensity to use urgent referral. Patients in these practices had a 7% increased mortality rate compared with those from practices with higher rates of urgent referral. For practices that have a consistently low propensity to use the urgent referral pathway (for example, on measures and in consecutive years), the data suggest that an increased use could plausibly lead to lower mortality and higher survival of patients with cancer.

The absolute differences in cumulative mortality are around 5% or 6% points in the risk of death within four years after the cancer diagnosis. This difference approaches the magnitude of known and important differences in survival between England and comparable countries^41^ or between socioeconomic groups within England.^42^

**What is already known on this topic**

The urgent referral pathway for patients with suspected cancer has been available in England since the early 2000s, but its impact on cancer survival is unknown

**What the paper adds**

We analysed survival of 215 284 patients with cancer in England, in relation to their general practice’s propensity to use urgent referral
The propensity to use the urgent referral pathway was associated with reduced mortality
General practices that consistently have a low propensity to use urgent referrals could consider increasing their use of this pathway, thereby plausibly increasing the survival of their patients with cancer

## Notes

The English national Cancer Waiting Times database was obtained from NHS England, containing data from the National Cancer Waiting Times Monitoring Dataset.

Ethical approval: Ethics committee approval not required for research based on routine data. Approval according to section 251 of the NHS Act 2006 applies to cancer registration and cancer intelligence in Public Health England.

Data sharing: The analysis is based on routine data from the NHS and Public Health England. No additional data are available from the authors.

The lead author (the manuscript’s guarantor) affirms that the manuscript is an honest, accurate, and transparent account of the study being reported; that no important aspects of the study have been omitted; and that any discrepancies from the study as planned (and, if relevant, registered) have been explained.

## Notes

Cite this as: *BMJ* 2015;351:h5102
