# Supplementary material for: Use of the English urgent referral pathway for suspected cancer and mortality in patients with cancer: cohort study
Source: BMJ. 2015 Oct 14;351:h5102. doi: 10.1136/bmj.h5102 (PMC4604216; doi:10.1136/bmj.h5102)

Web figure: HRs for death in relation to referral ratio, conversion rate and detection rate deciles, adjusted for age, sex and cancer type, and mutually adjusted

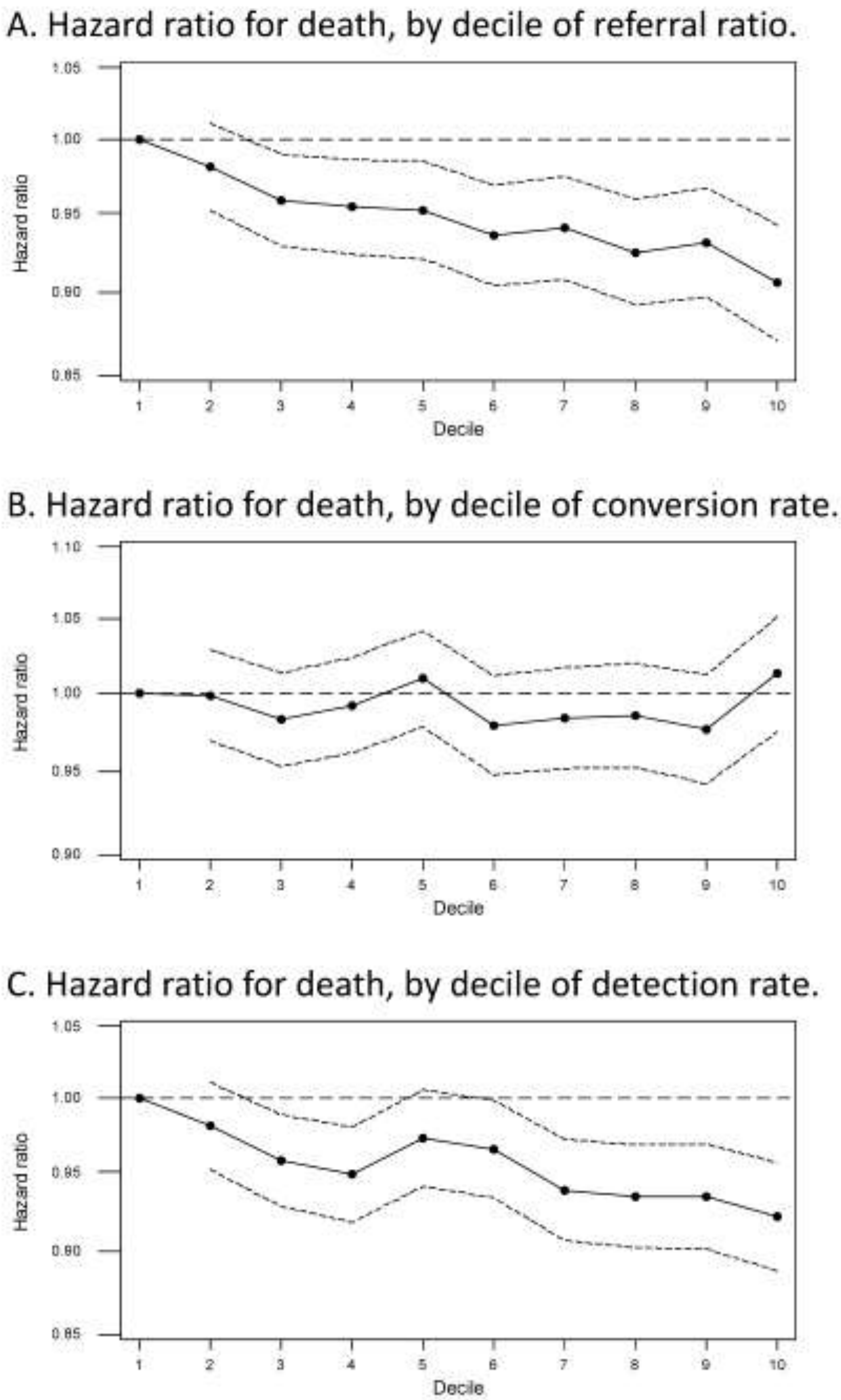

Supplement: Supplementary file 1 [file molh025004.wf_default.pdf]
