# Supplementary material for: Use of the English urgent referral pathway for suspected cancer and mortality in patients with cancer: cohort study
Source: BMJ. 2015 Oct 14;351:h5102. doi: 10.1136/bmj.h5102 (PMC4604216; doi:10.1136/bmj.h5102)
Supplement: Supplementary file 2 [file molh025004.ww_default.pdf]

Appendix. Analysis of the three main cancer patient groups identified in Figure 3 on the basis of GP urgent referral characteristics. Stratified analyses by cancer type and sensitivity analyses with different durations of follow-up, inclusion criteria and regression model specifications.

A. Overall analysis (215,284 patients; 91,620 deaths)

Group*	Patients	%	Deaths	HR	95% CI	
1	34,758	16	14,355	0.96	0.94	0.98
2	101,110	47	42,621	1.00		
3	79,416	37	34,644	1.07	1.05	1.08
p-trend				*<0.001*		

B. Restricted to colorectal cancer

Group	Patients	%	Deaths	HR	95% CI	
1	3,865	16	1,670	0.97	0.92	1.02
2	11,611	47	5,175	1.00		
3	9,160	37	4,145	1.03	0.99	1.08
p-trend				*0.017*		

C. Restricted to lung cancer

Group	Patients	%	Deaths	HR	95% CI	
1	3,596	16	3,107	0.96	0.93	1.00
2	10,411	46	9,086	1.00		
3	8,779	39	7,743	1.05	1.02	1.08
p-trend				*<0.001*		

D. Restricted to female breast cancer

Group	Patients	%	Deaths	HR	95% CI	
1	5,108	15	997	1.05	0.98	1.13
2	15,964	48	2,872	1.00		
3	12,497	37	2,172	1.03	0.97	1.09
p-trend				*0.842*		

E. Restricted to prostate cancer

Group	Patients	%	Deaths	HR	95% CI	
1	4,325	17	984	0.95	0.88	1.02
2	12,033	47	2,826	1.00		
3	9,297	36	2,239	1.07	1.01	1.13
p-trend				*0.001*		

F. Restricted to cancers other than colorectal, lung, breast, prostate.

Group	Patients	%	Deaths	HR	95% CI	
1	17,827	16	7,581	0.94	0.92	0.97
2	50,976	47	22,632	1.00		
3	39,601	37	18,324	1.09	1.07	1.11
p-trend				*<0.001*		

G. All cancers excluding female breast cancer

Group	Patients	%	Deaths	HR	95% CI	
1	29,650	16	13,358	0.95	0.93	0.97
2	85,146	47	39,749	1.00		
3	66,919	37	32,472	1.07	1.05	1.09
p-trend				*<0.001*		

H. Follow-up time limited to 0-1 year after diagnosis

Group	Patients	%	Deaths	HR	95% CI	
1	34,758	16	7,842	0.94	0.92	0.97
2	101,110	47	23,764	1.00		
3	79,416	37	20,000	1.09	1.07	1.11
p-trend				*<0.001*		

I. Follow-up time limited to 0-2 year after diagnosis

Group	Patients	%	Deaths	HR	95% CI	
1	34,758	16	11,020	0.96	0.94	0.98
2	101,110	47	32,867	1.00		
3	79,416	37	27,146	1.08	1.06	1.09
p-trend				*<0.001*		

J. Also adjusted for the patient's socioeconomic status**

Group	Patients	%	Deaths	HR	95% CI	
1	34,758	16	14,355	0.96	0.94	0.98
2	101,110	47	42,621	1.00		
3	79,416	37	34,644	1.06	1.05	1.08
p-trend				*<0.001*		

K. Diagnosis and length of follow-up consistent with cancer register

Group	Patients	%	Deaths	HR	95% CI	
1	27,112	16	10,913	0.95	0.93	0.98
2	78,371	47	32,258	1.00		
3	60,664	37	25,795	1.06	1.05	1.08
p-trend				*<0.001*		

L. Not adjusted for type of cancer

Group	Patients	%	Deaths	HR	95% CI	
1	34,758	16	14,355	0.97	0.95	0.99
2	101,110	47	42,621	1.00		
3	79,416	37	34,644	1.08	1.06	1.09
p-trend				*<0.001*		

M. Also adjusted for GP population age and sex distribution***

Group	Patients	%	Deaths	HR	95% CI	
1	34,758	16	14,355	0.97	0.95	0.99
2	101,110	47	42,621	1.00		
3	79,416	37	34,644	1.05	1.04	1.07
p-trend				*<0.001*		

N. Shared frailty model for prostate cancer (GP as random effect)

Group	Patients	%	Deaths	HR	95% CI	
1	4,325	17	984	0.95	0.88	1.02
2	12,033	47	2,826	1.00		
3	9,297	36	2,239	1.07	1.01	1.13
p-trend				*0.001*		

*Group 1: High referral ratio and high detection rate.
Group 2: All other combinations than group 1 and group 3.
Group 3: Low or low/intermediate referral ratio and detection rate
Hazard ratios adjusted for age, sex and cancer type.

**From J: Most vs. least deprived quintile:
HR: 1.34 (1.32-1.37); p-trend over quintiles <0.001
141 records with missing value for SES included as a separate category

***From M: Effects of GP population characteristics:
HR: 0.88 (0.86-0.90) for highest vs. lowest tertile of median age
HR: 1.13 (1.08-1.17) per 10 percentage point increase in male proportion
